# The optimal management of Seymour fractures in children and adolescents: a systematic review protocol

**DOI:** 10.1186/s13643-020-01407-5

**Published:** 2020-06-23

**Authors:** Ailbhe L. Kiely, Grant S. Nolan, Lilli R. L. Cooper

**Affiliations:** 1 Royal Stoke University Hospital, Stoke-on-Trent, UK; 2 Division of Surgery and Interventional Science, Royal Free Hospital, University College London, London, UK; 3 St Thomas’ Hospital, London, UK

**Keywords:** Seymour fracture(s), Juxta-epiphyseal fracture(s), Infection, Osteomyelitis, Complication(s)

## Abstract

**Background:**

Seymour fractures are open, displaced juxta-epiphyseal fractures of the distal phalanx, with an overlying nail bed laceration that occur in children and adolescents with an open physis. This fracture occurs rarely, but its potential consequences are clinically significant. Due to anatomical particulars and proximity to the growth plate, this open fracture may result in soft tissue infection and osteomyelitis, leading to growth arrest and persistent mallet deformity. At present, there is no consensus as to the optimal management of Seymour fractures. The objective of this study will be to systematically evaluate the existing evidence on the management of Seymour fractures in children and adolescents and to establish what are the most important factors pertaining to an uncomplicated recovery.

**Methods:**

We designed and registered a study protocol for a systematic review of randomised controlled trials and observational studies. A comprehensive literature search will be conducted (from inception to present) in MEDLINE, EMBASE, CINAHL and Cochrane Library databases. Grey literature will be identified through searching Open Grey and dissertation databases using an exhaustive search strategy. All clinical studies examining the management of Seymour fractures will be included. The interventions (irrigation and debridement; prophylactic antibiotics) and their timings (early vs late) will be compared to no antibiotics and no debridement. Primary outcome measures will be the incidence of superficial and deep infection. Secondary outcomes will include other adverse events such mal-union, non-union, need for re-operation, physeal disturbance and nail dystrophy/atrophy. Two independent reviewers will screen all citations, full-text articles, and abstract data. Conflicts will be resolved through discussion. The study methodological quality (or bias) will be appraised using an appropriate tool. A narrative synthesis will be performed. If data permits, we will conduct random-effects meta-analysis where appropriate.

**Discussion:**

This review will provide evidence for the management of Seymour fractures, based on a cumulation of existing smaller studies. Due to the rarity of this fracture pattern, included studies are expected to be mainly observational and prone to bias; however, there is value in summarising the evidence to guide clinicians.

**Systematic review registration:**

Systematic review registration: PROSPERO CRD42020153726.

## Background

Seymour fractures are displaced, open fractures of the juxta-epiphyseal region of the distal phalanx [1], with an overlying nail bed laceration that occur in skeletally immature individuals. While this fracture pattern was first described by Seymour in 1966 [1], the exact definition is not unanimous; the majority of sources [2–11] define it as an open injury; however, several sources also include closed injuries [12, 13]. Seymour’s original description of the fracture did not specifically comment on the presence or absence of nail bed injury, and thus, the definition is subject to interpretation.

Additionally, some sources identify a similar injury pattern in adults and include this in their definition [14]. Radiologically, these can be fractures of the epiphysis (Salter-Harris types I and II) or metaphyseal fractures just distal to the physis (juxta-epiphyseal). Salter-Harris III–V are generally not included in the definition as these either cross the epiphyseal plate or would not cause the same displacement or clinical pseudo-mallet deformity [3]. Clinically, they may mimic a mallet type injury due to the insertion points of the flexor digitorum profundus and the opposing extensor tendon—causing a deformity where the shaft of the distal phalanx is flexed and the epiphysis remains extended [12].

### Disease burden, morbidity in general

The incidence of Seymour fractures has never been reported, such is the rarity of the fracture. In 2020, Rask described a local prevalence of 5.4% of all paediatric distal phalanx fractures presenting to one institution [15]. More broadly speaking, the annual occurrence of a phalangeal fracture is 2.7% in children [16]. Seymour fractures most commonly occur in younger children, with a reported mean age of 8.7 years [17]. The most common mechanism is a crush or sporting injury [4].

While these are rarely occurring fractures, the clinical impact and consequences of them are significant. Despite appearing as a potentially insignificant injury, these fractures are high risk for complications and cause a disproportionately large amount of morbidity.

### Consequences of infection in Seymour fractures

Owing to several factors, this fracture is high risk for infection [12, 17]. Late presentation or lack of intervention may result in infection, growth arrest and persistent mallet deformity of the distal phalanx [10]. Reyes reported a 45% overall infection rate with a 36% occurrence of osteomyelitis with presentation > 24 h post-injury [17]. The risk of infection is higher than in other open fractures of the distal phalanx due to the characteristic soft tissue injury. The nail plate is avulsed, and interposed soft tissue, the germinal matrix of the nail complex, may be present in the fracture site, leading to contamination of the fracture site [18]. In turn, an infection in the juxta-epiphyseal region of bone can lead to physeal arrest.

These injuries are also high risk for non-union [12]. In the same vein, this is postulated to be due to their unique anatomical considerations. In a juxta-epiphyseal fracture such as this, the extensor tendon inserts onto the proximal segment of the fracture and the flexor tendon to the distal one [1, 18], so forces across the fracture oppose union [11]. Any interposed nail bed as previously described can also prevent union [17].

A growth arrest of the distal phalanx, whether caused by infection or mal-union, has the potential to alter the normal arcade of finger lengths and result in cosmetic deformity [10].

### Current practice

A range of different management options have been reported in a variety of different settings [5, 17]. These range from closed reduction and splinting to formal washout, debridement and percutaneous Kirshner wire (k-wire) fixation. These interventions may take place in clinic, the emergency department (A&E) [5] or the operating theatre [3, 17] under local anaesthetic (ring block) [5] or general anaesthesia [3, 17]. The current practice in the management of Seymour fractures varies significantly amongst different surgeons and centres.

The rationale for conservative management is based on the principal of metalwork increasing the risk of infection, and from Seymour’s original case series of this fracture pattern, where he describes high rates of post-operative infections [1, 19]. Seymour found a higher incidence of infection (3/5) in those managed operatively (k-wire) and those who had the nail-plate removed (3/6) than those who had a closed reduction and splint (0/9) [1]. This said, in Seymour’s original study, perioperative antibiotics were not given nor did ‘formal irrigation and debridement’ occur, as described by more recent studies [3].

The rationale for formal irrigation and debridement and prophylactic antibiotics is based on principles of the management of an open fracture and is a more widely accepted practice in more recent years [3, 7, 12, 13, 18].

Nonetheless, with a paucity of evidence informing the management of these fractures, equipoise exists.

### Hypothesis/aims

We hypothesize that Seymour fractures that undergo timely formal irrigation, debridement and reduction with early prophylactic antibiotics have a lower rate of complications such as infection and mal-union. This systematic review aims to summarise the best available evidence for the management of Seymour fractures in children and adolescents. This review will be directly applicable to the clinical care of these injuries and will provide higher-level evidence for their management. This is of clinical relevance, in a fracture that is high risk for complications, which may be avoided when managed with early appropriate care [5, 17].

### Research question

In children and adolescents who sustain Seymour fractures, what are the most important factors pertaining to an uncomplicated recovery?

## Materials and methods

This protocol has been registered with the PROSPERO international prospective register of systematic reviews (registration number CRD42020153726) and will be reported adhering to the Preferred Reporting Items for Systematic Review and Meta-Analysis Protocols (PRISMA-P) 2015 statement [20]. The PRISMA-P checklist for this study is included as an additional file (Supplementary file 1).

The final review will be reported following the PRISMA statement [21] and the Meta-Analysis of Observational Studies in Epidemiology (MOOSE) guidelines [22].

### Study eligibility criteria

Studies will be selected according to participants, condition or outcome(s) of interest and study design.

#### Study designs

We will include randomized controlled trials (RCT) and controlled observational studies assessing the management of Seymour fractures. We expect the majority of studies to be retrospective or prospective observational studies (cohort or case-control) with or without a comparative group. Due to the anticipated paucity of comparative studies, we will also include non-comparative studies, e.g., case series, with intention of pooling outcomes with single-arm data from comparative studies into a meta-analysis of proportions. Case series of less than 3 patients, case reports, study protocols, animal studies and review articles will be excluded.

#### Participants

We will include studies examining the management of Seymour fractures in children and adolescents, where these are persons aged under 18 years with open physeal plates. Studies reporting adults (aged over 18) or any persons with fused epiphyseal plates will be excluded, as will patients without radiological confirmation of a juxta-epiphyseal fracture. Closed injuries will also be excluded.

#### Interventions and comparators

We will broadly group patients based on two treatment modalities, namely debridement and prophylactic antibiotics and the timing of these interventions. Debridement will be defined as formal washout, debridement of soft tissues and any form of splinting or fixation. This may take place in the emergency department, clinic room or operating theatre.

Antibiotics may be in the oral or intravenous form and must be prescribed from time of presentation (< 24 h).

To establish the isolated and combined importance of early debridement and prophylactic antibiotics, we will compare the following:

Early, late or no formal debridement ± reduction and fixation as needed;

Early vs late or no antibiotics.

Early antibiotics will be defined as prophylactic antibiotics administered from the time of presentation, as long as presentation was within 24 h of injury.

Early debridement eludes to debridement within 48 h of injury.

In addition, these will be compared in combination as ‘complete’ vs ‘incomplete’ treatment, where complete treatment is defined as a combination of early antibiotics and debridement ± fracture reduction and fixation if appropriate. Incomplete treatment will be defined as either a lack of debridement ± reduction and splinting or fixation or a lack of early prophylactic antibiotics but the presence of the other. No treatment pertains to a lack of both components.

For those who underwent debridement, a subgroup comparison will be conducted, examining emergency department vs operating theatre management. Those patients who were delayed presenting to medical care will also be analysed as a separate group. If reported, usual care such as pain relief, anaesthesia and immobilisation technique will be examined in addition.

#### Outcome measures

The primary outcome will be the relative risk of soft tissue or bony infection. Soft tissue infection is defined as those with characteristic signs of skin and subcutaneous tissue infection (erythema, warmth, purulence). Bony infection (osteomyelitis) is defined as those that had signs of infection combined with radiographic evidence of focal bony lysis or cortical loss or a periosteal reaction.

Secondary outcomes will include other adverse events such mal-union, non-union, need for re-operation, physeal disturbance and nail dystrophy/atrophy (all as defined by the study in question).

Mal-union and non-union will be assessed up to 1 year; nail growth and physeal disturbance will be assessed with a minimum follow-up of 3 months post-injury.

### Setting

Studies performed in the hospital and emergency department setting will be included. Studies performed in a primary care setting will be excluded.

### Language

No limitations will be imposed on language.

### Information sources

The primary source of literature will be a structured search of the following major electronic databases from inception to April 2020: MEDLINE (Ovid) SP; EMBASE (Ovid SP); CINAHL (Cumulative Index to Nursing and Allied Health Literature) and the Cochrane Library (Cochrane Database of Systematic Reviews, Cochrane Central Register of Controlled Trials [CENTRAL], Cochrane Methodology Register), in collaboration with a medical research librarian. PROSPERO will be searched for ongoing or recently completed systematic reviews.

The secondary source of potentially relevant material will be a search of the grey or difficult to locate literature, including Open Grey and dissertation databases (e.g. Open Access Theses and Dissertations). We will hand-search and screen the reference lists of included studies, relevant reviews, national clinical practice guidelines or other relevant documents to identify cited articles not already in our list of included studies. Content experts and authors who are prolific in the field will be contacted. The literature searches will be designed and conducted by the review team which includes two experienced health information specialists.

### Search strategy

The search strategy used will include a range of text words as well as Medical Subject Headings (MeSH) terms related to ‘Seymour fractures’ and ‘juxta-epiphyseal fractures.’ The draft search strategy for MEDLINE is presented in Supplementary file 2. These search terms will be adapted for use with other bibliographic databases.

No restrictions will be placed on the timing of publication. The search will be performed in English, and translations will be sought for articles published in other languages. No restriction will be placed on publication status (i.e. unpublished studies will be included).

### Selection of studies

Once the text and MeSH searches have been combined, duplicates will be removed using EndNote (Clarivate Analytics, Boston, MA, USA). Citations will also be managed using this software.

The collated reference list of studies meeting the inclusion criteria will be searched to identify additional relevant studies. Two independent researchers (A.K. and G.N.) will screen titles and abstracts for eligibility against a pre-defined list of inclusion and exclusion criteria. This process will be carried out using Rayyan [23], a bespoke web and mobile app for systematic reviews. At this stage, any reference deemed eligible for inclusion by either reviewer will be included. Two reviewers (A.K. and G.N.) will then screen the full text of potentially relevant articles. Reasons for exclusion will be recorded where applicable.

Where disparity occurs between references, consensus will be sought, and all remaining articles will be read in full before a decision on inclusion is made. If disagreements remain between the screening authors, the texts will be resolved by discussion or by consulting a third author (L.C.).

The bibliography of the final included studies will be screened to check for additional publications that may be relevant. The search results, including abstracts, full-text articles and record of the reviewer’s decisions will be recorded first in Rayyan [23] and then in a pre-defined data collection sheet in Microsoft Excel (Microsoft Corporation, 2018).

### Data extraction and management

Two reviewers (A.K. and G.N.) will collect data independently and in duplicate using a pre-defined electronic data extraction form. The data collection process will be in keeping with the Cochrane Handbook of Systematic Reviews of interventions [24].

The following data will be extracted: first author, year of publication, study design, inclusion and exclusion criteria, number of patients, method of diagnosis, age, sex, relevant medical history, mechanism, time since injury, digit involved, type of intervention (debridement, fixation, anaesthesia), duration of intervention, specialty performing intervention, location of intervention (theatre or A&E), antibiotic regimen, time to first antibiotic, analgesic regimen and primary and secondary outcomes.

If authors report on adult patients, these will not be included in the analysis, if the data is clearly distinguishable.

In addition, the statistical analysis models and outcome measures used will be noted. Divergences will be resolved by consensus or with a third reviewer (L.C.) if needed.

### Dealing with missing data

Where relevant, study authors will be contacted if data relevant to the systematic review are missing in the study report. Where authors fail to reply after first contact or after one reminder, the missing data will be acknowledged, and we will proceed with the analyses.

### Assessment of risk of bias of included studies

We expect that most included studies will be observational rather than randomised studies, of which some will be uncontrolled (e.g. case series of one surgeon’s outcomes). Each study design will be assessed using a relevant tool.

If there are any randomised trials, they will be assessed using the Cochrane Risk of Bias 2 (RoB 2) [25] tool, which focuses on aspects of trial design, conduct and reporting.

Non-randomised comparative studies (e.g. cohort and case control studies) will be assessed using ROBINS-I tool [26], which holds studies to standard against a hypothetical pragmatic randomised trial. It covers seven potential domains of bias.

Uncontrolled studies (e.g. case series) will be assessed using a tool which has been specifically for this purpose [27]. It is formed from an adaptation of previous criteria from Pierson [28], Bradford Hills [29] and Newcastle Ottawa scale [30] which assess bias in four domains: selection, ascertainment, causality and reporting.

A narrative summary of the risk of bias of the included studies will be performed, which will be supported by a figure and table showing the results of the critical appraisal. The results of the risk of bias tool will be used in a sensitivity analysis to ensure studies judged to be at ‘critical’ risk of bias do not affect the robustness of our results in any subsequent meta-analysis.

### Data analysis and synthesis

To answer the review question of determining the optimal management of Seymour fractures, the data from each paper will be used to build evidence tables providing an overall description of included studies. The tables will contain data including study characteristics, context, population, outcomes and findings for each included study. This will be accompanied by a narrative synthesis of the data.

Clinical and methodological heterogeneity will be assessed across each study in early vs late debridement, early vs late antibiotics and complete vs incomplete treatment groups [31]. This will determine whether it may be feasible to perform a meta-analysis. If possible, we will perform a random effects meta-analysis [31]. We will present the results as a pooled estimate for each of the primary outcomes comparing early vs late debridement, early vs late antibiotics and complete vs incomplete management as relative risk with 95% confidence intervals. The results of this will be presented in a forest plot. If feasible and appropriate, studies that do not contribute comparative data will be pooled with single-arm data from comparative studies to determine the overall risk of infection in the treatment group of relevance by meta-analysis of proportions. A forest plot may be produced to show the pooled effect of findings.

Heterogeneity will be assessed visually by examining the overlap of confidence intervals in the forest plot. We will quantify statistical heterogeneity by estimating the variance between studies using the *I*^2^ statistic which examines the variance between studies to produce a percentage score of between 0 and 100% which will be interpreted as per the Cochrane handbook [24]. Tau squared and chi-squared tests will also be applied where a *P* value of < 0.05 is considered statistically significant for heterogeneity [31].

A summary of findings table will be created for the primary outcome measure. We will rate the overall quality of evidence of these outcomes using the Grading of Recommendations, Assessment, Development and Evaluation (GRADE) Working Group methodology [32]. Each critical outcome’s quality of evidence is rated, taking into consideration five defined criteria (risk of bias and limitations of design, consistency of analysed studies and their results, directness, precision and publication bias) that may lead to grading down, and three criteria (large effect, dose-response and opposing bias and confounders) that may lead to grading up [33, 34].

### Additional analyses

If it is not possible to combine the data in the above manner, then we will determine the crude incidence estimates of infection (number of infections/sample size) along with the 95% confidence intervals associated with timing of debridement and prophylactic antibiotics for each study using a meta-analysis of proportions.

If sufficient studies are identified and data points are available, potential sources of heterogeneity will be investigated further by subgroup analyses according to baseline characteristics and methodological covariates. We plan to conduct analyses by gender (male vs female), age (children vs adolescents) and risk of bias (e.g. serious vs moderate/low risk of bias). A subgroup analysis will be carried out of patients who were delayed presenting to hospital, as a meta-analysis of proportion to ascertain the incidence of infection in this cohort. For all patients who underwent debridement, a subgroup comparison will be conducted, examining emergency department vs operating theatre management.

### Meta-bias

Publication bias will be investigated, and a funnel plot will be generated for each meta-analysis containing 10 or more studies. Small study effects (or ‘publication bias’ across studies) will be assessed by inspection of the funnel plots for asymmetry and with Egger’s test [35] where appropriate, with the results considered to indicate potential small study effects when *P* values are < 0.10. Depending on the number of included studies in the review, we will undertake a sensitivity analysis to ensure the robustness of our results. We anticipate that the systematic review will identify studies judged to be at critical risk of bias, and we will perform a sensitivity analysis where these are excluded.

## Discussion

This protocol describes a systematic review and meta-analysis of the management of Seymour fractures in children and adolescents. We are not aware of another systematic review addressing this cohort of patients.

While the hand is the most frequently injured part of a child’s body, Seymour fractures are relatively uncommon injuries [36]. While we can ascertain certain aetiological details from studies, review articles and case series [5, 11, 12, 18], the paucity of evidence due to the rarity of this fracture pattern leads to controversy as to the optimal type and setting of treatment.

A key challenge arising from the rarity of the fracture is that we anticipate identifying studies that are small in size, use diverse designs and have variable quality of reporting methods and results. We expect to find few comparative studies, and all studies will likely be retrospective cohort studies. By design, they will carry a high risk of bias. As such, bias will be a potential limitation of this review. We also expect the small number of studies to limit the potential for meta-analysis of studies although we will continue to proceed with a narrative review in this instance.

Any amendments made to this protocol when the review is conducted will be reported in the final paper. We plan to publish the review in a peer-reviewed journal that will reach an audience of both orthopaedic and plastic surgeons. We also anticipate that our findings will be of interest to paediatric emergency practitioners, paediatric surgeons or patients or parents who have sustained Seymour fractures.

This study aims to identify the treatment setting and modality that offers the best outcome for Seymour fractures in a comparison of several studies, and therefore provide clinicians with information to choose the optimal treatment plan for these rare fractures. Our conclusions will be based on validated methodology, including a quality of evidence and quality of reporting appraisal for each study.

When summarising the results for this important clinical presentation, this systematic review will help guide clinicians in improving the management of these high-risk injuries.

## Supplementary information

**Supplementary file 1.** PRISMA-P checklist.

**Supplementary file 2.** Draft search strategy for Medline.

## Data Availability

Not applicable.

## Abbreviations

A&E: Accident and emergency
CENTRAL: Cochrane Central Register of Controlled Trials
CINAHL: The Cumulative Index to Nursing and Allied Health Literature
EMBASE: Excerpta Medica Database
GRADE: Grading of Recommendations, Assessment, Development and Evaluation
MA: Massachusetts
MEDLINE: Medical Literature Analysis and Retrieval System Online
MeSH: Medical Subject Headings
MOOSE: Meta-Analysis of Observational Studies in Epidemiology
PRISMA: Preferred Reporting Items for Systematic Review and Meta-Analysis
PRISMA-P: Preferred Reporting Items for Systematic Review and Meta-Analysis Protocols
PROSPERO: International prospective register of systematic reviews
RCTs: Randomised controlled trials
ROBINS-I: Risk of Bias In Non-randomized Studies - of Interventions
USA: United States of America
