# Supplementary material for: The optimal management of Seymour fractures in children and adolescents: a systematic review protocol
Source: Syst Rev. 2020 Jun 23;9:150. doi: 10.1186/s13643-020-01407-5 (PMC7313162; doi:10.1186/s13643-020-01407-5)
Supplement: Supplementary file 2 — Draft search strategy for Medline. [file 13643_2020_1407_MOESM2_ESM.docx]

| **#** | **Database** | **Search term** |
| --- | --- | --- |
| 1 | Medline | (seymour ADJ1 fracture*).ti,ab,af |
| 2 | Medline | ((((juxtaepiphyseal OR juxta-epiphyseal) AND phalanx) AND (distal OR terminal)) AND fracture*).ti,ab |
| 3 | Medline | (1 OR 2) |

**Additional file 2 – Draft search strategy for Medline**
